# Clinical impact of intratumoral HER2 heterogeneity on trastuzumab efficacy in patients with HER2-positive gastric cancer

**DOI:** 10.1007/s00535-018-1464-0

**Published:** 2018-04-09

**Authors:** Takeru Wakatsuki, Noriko Yamamoto, Takeshi Sano, Keisho Chin, Hiroshi Kawachi, Daisuke Takahari, Mariko Ogura, Takashi Ichimura, Izuma Nakayama, Hiroki Osumi, Tomohiro Matsushima, Mitsukuni Suenaga, Eiji Shinozaki, Naoki Hiki, Yuichi Ishikawa, Kensei Yamaguchi

**Affiliations:** 1 Department of Gastroenterology, The Cancer Institute Hospital of Japanese Foundation for Cancer Research, 3-8-31 Ariake, Koto-ku, Tokyo, 135-8550 Japan; 2 Department of Pathology, The Cancer Institute Hospital of Japanese Foundation for Cancer Research, Tokyo, Japan; 3 Department of Gastric Surgery, The Cancer Institute Hospital of Japanese Foundation for Cancer Research, Tokyo, Japan

**Keywords:** Intratumoral HER2 heterogeneity, Trastuzumab, Predictive marker, Chemotherapy, Gastric cancer

## Abstract

**Background:**

There is growing interest in the clinical significance of intratumoral HER2 heterogeneity. Its prognostic and predictive impacts on trastuzumab efficacy were demonstrated in breast cancer. However, its clinical significance in gastric cancer is still unclear.

**Methods:**

Twenty-eight HER2-positive gastric cancer patients who had gastrectomy prior to trastuzumab-based chemotherapy were consecutively enrolled. Intratumoral HER heterogeneity was evaluated using whole-tissue sections by immunohistochemistry. When all tumor cells overexpressed HER2 protein, the tumor was defined as homogeneously HER2 (Homo-HER2)-positive group. The others were defined as heterogeneously HER2 (Hetero-HER2)-positive group.

**Results:**

There was no significant difference in clinicopathological features between the two groups. The median progression-free survival (PFS) and overall survival (OS) in the Homo-HER2-positive group were significantly longer than those in the Hetero-HER2-positive group (PFS; 20.0 months [95% CI 17.8–22.2] vs. 6.0 months [95% CI 2.3–9.7]; HR 0.11; 95% CI 0.03–0.41; *p* < 0.001, OS; not reached vs. 14.0 months [95% CI 11.9–16.1]; HR 0.18; 95% CI 0.06–0.61; *p *= 0.003). In the multivariate analysis, these associations remained significant both in PFS (HR 0.12; 95% CI 0.03–0.46, *p* = 0.002) and OS (HR 0.21; 95% CI 0.06–0.72, *p* = 0.013). With respect to response rate, no statistical difference was found between two groups. However, deeper tumor shrinkage was obtained in the Homo-HER2-positive group compared with the Hetero-HER2-positive group (*p* = 0.046).

**Conclusions:**

Intratumoral HER2 heterogeneity may have robust clinical impact on trastuzumab efficacy in patients with HER2-positive gastric cancer. These findings should be validated by larger independent cohorts and further molecular correlative analyses are warranted.

**Electronic supplementary material:**

The online version of this article (10.1007/s00535-018-1464-0) contains supplementary material, which is available to authorized users.

## Introduction

Gastric cancer is currently the third-most common cause of cancer-related deaths and the fifth-most common diagnosed cancer worldwide [1]. Endoscopic resection, standard gastrectomy with D2 lymphadenectomy, and multidisciplinary therapy have improved outcomes [2–5]. However, the prognosis for inoperable or metastatic gastric cancer is still poor and median overall survival (mOS) is less than 1 year globally [6, 7].

Human epidermal growth factor receptor 2 (HER2) is involved in tumor proliferation, differentiation, and survival [8]. Its amplification or protein overexpression is shown in up to 20% of cases in gastric cancer [9–11], suggesting that it is a critical driver oncogene and, therefore, a promising treatment target. Trastuzumab, full humanized anti-HER2 antibody, has been successfully developed in HER2-positive breast cancer [12, 13]. In gastric cancer, the ToGA trial showed significant clinical benefits of the addition of trastuzumab to standard chemotherapy [14]. Inclusion criteria of the ToGA trial were initially designed as HER2 immunohistochemistry (IHC) 3+ or fluorescence in situ hybridization (FISH) positive. But post hoc analysis showed that HER2 IHC 3+ or IHC 2+/FISH-positive groups benefited more from trastuzumab than the other groups, leading to different indications for trastuzumab use across the world. In the US, trastuzumab was approved for IHC 3+ or FISH positive. Meanwhile, in Europe and Japan, trastuzumab use is limited in IHC 3+ or IHC 2+/FISH positive.

Trastuzumab-based first-line treatment now represents a standard approach. However, its treatment efficacy varies among patients; the overall response rate is widely reported with frequency from 47.0 to 68.0% [14–16]. This indicates that there is a considerable proportion of patients refractory to trastuzumab and the present criteria for trastuzumab use may be insufficient to predict the response to trastuzumab. Therefore, a new biomarker which can predict trastuzumab efficacy is needed.

There is growing interest in intratumoral HER2 heterogeneity in breast and gastric cancer. Compared with breast cancer, HER2 heterogeneity in gastric cancer has been frequently recognized from 39.0 to 75.4% evaluated by IHC [17–21] and occasionally becomes a problem for assessment of HER2 [22]. Clinical significance of intratumoral HER2 heterogeneity as a predictive factor for trastuzumab was reported in breast cancer; namely, homogeneously HER2 overexpressed breast cancer showed more benefit from trastuzumab-based treatment compared with heterogeneously HER2 overexpressed breast cancer [23]. However, its clinical significance in gastric cancer is still unclear [24].

We hypothesized that, as in HER2-positive breast cancer, intratumoral HER2 heterogeneity may have clinical impact on trastuzumab efficacy in HER2-positive gastric cancer. To test our hypothesis, we evaluated intratumoral HER2 heterogeneity within a tumor and investigated its clinical relevance to trastuzumab efficacy in patients with HER2-positive advanced gastric cancer.

## Methods

### Patients

One-hundred and thirty-one patients with histologically confirmed HER2-positive advanced or metastatic gastric cancer were treated by trastuzumab-based chemotherapy as first-line treatment in The Cancer Institute Hospital of Japanese Foundation for Cancer Research from March 2011 to December 2015. Among them, 28 patients, who had previous gastrectomy and whose primary tumor samples were available, were consecutively enrolled to evaluate intratumoral HER2 heterogeneity in the whole-tissue sections. Computed tomography assessments were repeated every 6–8 weeks and RECIST ver1.1 was used to define all responses. Clinical information was retrospectively collected from medical records. This study was approved by the Institutional Review Boards (No. 2015-1029) and all patients signed an informed consent for the analysis of molecular correlates.

### Treatment schedule

Chemotherapy was done in accordance with the ToGA trial [14]. Briefly, cisplatin 80 mg/m^2^ on day 1 was given by intravenous infusion and it was given every 3 weeks until six cycles. Capecitabine 1000 mg/m^2^ was given orally twice a day for 14 days followed by a 1-week rest. Trastuzumab was given by intravenous infusion at a dose of 8 mg/kg on day 1 of the first cycle, followed by 6 mg/kg every 3 weeks. Treatments were continued until disease progression or patient’s refusal or intolerable adverse effect despite appropriate dose reduction.

### Evaluation of HER2

A formalin-fixed paraffin-embedded block including the maximum invasive area of the tumor was selected in each patient and was cut into 4-μm section. IHC was conducted using an BenchMark ULTRA autostainer (Ventana, Tucson, AZ, USA) and the primary antibody used was anti-HER2 (4B5) (10798; rabbit monoclonal; Ventana, Tucson, AZ, USA) according to the manufacturer’s instructions. The HER2 protein overexpression was evaluated using the ToGA trial scoring system for surgical resected gastric cancer tissue obtained from primary lesion: 0, no reactivity or membranous reactivity in less than 10% of tumor cells; 1+, faint or barely perceptible membranous reactivity in at least 10% of tumor cells; 2+, weak to moderate complete, basolateral or lateral membranous reactivity in at least 10% of tumor cells; and 3+, strong complete, basolateral or lateral membranous reactivity in at least 10% of tumor cells [14]. When IHC score was 2+ by IHC, FISH was performed using Path Vysion HER2 DNA probe kit (Vysis/Abbott, Abbott Park, IL, USA) according to the manufacturer’s instructions. Briefly, the total numbers of HER2 and centromeric probe 17 (CEP17) signals were counted in at least 20 cancer cell nuclei in different two areas. When a HER2/CEP17 ratio was ≥ 2.2, it was diagnosed as HER2 gene amplification. IHC score of 3+ or IHC score of 2+ with FISH positivity was defined as HER2 positive, whereas IHC score of 0 or 1+, or IHC score of 2+ with FISH negativity was defined as HER2 negative.

### Evaluation of intratumoral HER2 heterogeneity

**Fig. 1:**
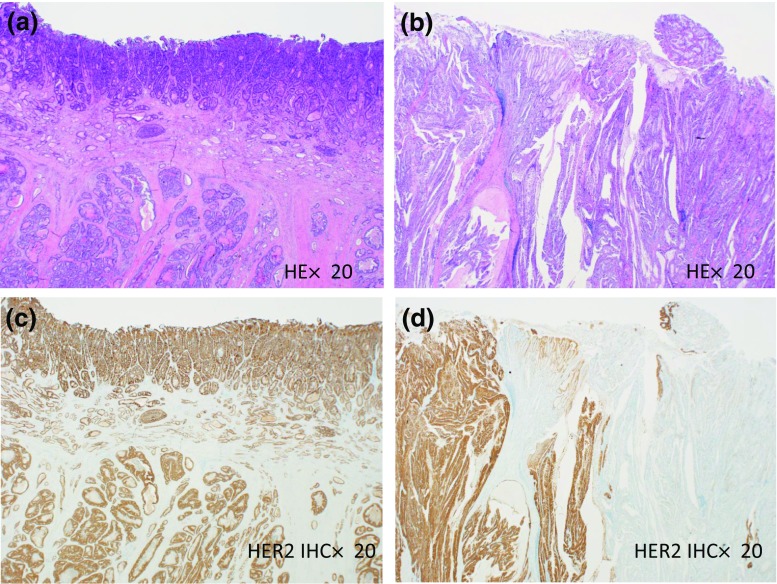
Representative images of homogeneously HER2 positive (**c**) and heterogeneously HER2 positive (d) in gastric cancer. Hematoxylin–eosin stain shows differentiated adenocarcinoma in **a** and **B** (× 20). HER2 IHC shows that almost all cancer cells overexpress HER2 protein in **c** (× 20). HER2 IHC shows heterogeneously HER2 overexpression: clearly demarcated HER2-positive area is recognized in left side while negative in right side in **d** (× 20)

Intratumoral HER2 heterogeneity was independently evaluated by two pathologists (NY and HK) without any clinical information. Because there is no guideline for the assessment of intratumoral HER2 heterogeneity in gastric cancer, we defined intratumoral HER2 heterogeneity as follows in this study; when all tumor cells on a section representing the tumor overexpressed HER2 protein with IHC 3+ or 2+, the tumor was defined as homogeneously HER2 (Homo-HER2)-positive group. The others were defined as heterogeneously HER2 (Hetero-HER2)-positive group. Representative images are shown in Fig. 1.

### Statistical analysis

Progression-free survival (PFS), OS, and objective response rates (ORRs) were evaluated. PFS was defined between the date of initial chemotherapy and first documented progression or death from any cause. OS was defined between the date of initial chemotherapy and death from any cause. If patients did not meet any endpoints until 31 December 2016, they were censored at the time of last contact. To evaluate the prognostic value of intratumoral HER2 heterogeneity, PFS and OS were estimated using Kaplan–Meier methods and compared by the log-rank test. Multivariate analysis using Cox proportional hazard model was performed by including covariates whose *p* value was less than 0.05 in univariate analysis. Patient characteristics and response rates were evaluated by Fisher’s exact test. Depth of response was compared using Mann–Whitney *U* test. The level of significance was set to *p *< 0.05 and all statistical tests were two sided. All statistical analysis was done using the SPSS ver.24 (SPSS Chicago, IL).

## Results

### Patient characteristics

Patient characteristics in this study are shown in Table S1. Male gender, stomach location, and differentiated histology were dominant in this cohort. More than half of patients had visceral (lung or liver) metastasis. Majority had received adjuvant chemotherapy using S-1, an oral fluoropyrimidine, for 1 year after the previous curative surgery [3]. None had any neoadjuvant chemotherapy prior to surgery. More than 90% of the patients were IHC 3+. Intratumoral HER2 heterogeneity was observed in half of the patients (50.0%). Concordance rate of diagnosis of HER2 heterogeneity between the two pathologists was 100%. Twenty-five patients were treated with capecitabine and cisplatin plus trastuzumab, while three patients were similarly treated without cisplatin due to old age. Table 1 shows a comparison of demographics between the two groups. The patients who were treated without cisplatin and the patients whose tumor was IHC 2+/FISH positive were all found in the Hetero-HER2-positive group; however, there was no significant difference in all characteristics between the two groups.

**Table 1:** Comparison of patients’ characteristics between Homo-HER2 and Hetero-HER2-positive groups in this study

Characteristics	Hetero-HER2 *n* = 14 (%)	Homo-HER2 *n* = 14 (%)	*p* values
Age	68.5	68.0	1.000
≥ 68	8 (57.1)	7 (50.0)	
< 68	6 (42.9)	7 (50.0)	
Gender			0.385
Male	12 (85.7)	9 (64.3)	
Female	2 (14.3)	5 (35.7)	
ECOG PS			1.000
0	8 (57.1)	8 (57.1)	
1–2	6 (42.9)	6 (42.9)	
Primary tumor site			1.000
GEJ	4 (28.6)	4 (28.6)	
Distal stomach	10 (71.4)	10 (71.4)	
Differentiation			0.326
Differentiated type	13 (92.9)	10 (71.4)	
Undifferentiated type	1 (7.1)	4 (28.6)	
Visceral metastasis			1.000
Yes	8 (57.1)	7 (50.0)	
No	6 (42.9)	7 (50.0)	
Adjuvant chemotherapy			1.000
Yes	8 (57.1)	9 (64.3)	
No	6 (42.9)	5 (35.7)	
Platinum-based			0.222
Yes	11 (78.6)	14 (100)	
No	3 (21.4)	0 (0.0)	
HER2 status			0.481
IHC 3+	12 (85.7)	14 (100)	
IHC 2+/FISH positive	2 (14.3)	0 (0.0)	
CEA			0.449
≥ 5.0 ng/ml	9 (64.3)	6 (42.9)	
< 5.0 ng/ml	5 (35.7)	8 (57.1)	
CA 19-9			1.000
≥ 37.0 U/ml	7 (50.0)	6 (42.9)	
< 37.0 U/ml	7 (50.0)	8 (57.1)	

*Hetero-HER2* heterogeneously HER2 positive, *Homo-HER2* homogeneously HER2 positive, *GEJ* gastroesophageal junction, *ECOG PS* Eastern Cooperative Oncology Group Performance Status, *FISH* fluorescence in situ hybridisation, *IHC* immunohistochemistry, *CEA* carcinoembryonic antigen, *CA 19-9* carbohydrate antigen 19-9

### Clinical impact of intratumoral HER2 heterogeneity on trastuzumab efficacy

The median follow-up period was 17.9 months for all patients and 33.0 months for the censored patients, respectively. The median PFS and OS were 11.0 months (95% CI 7.1–14.9) and 24.0 months (95% CI 4.6–43.4), respectively. Twenty-five patients (89.3%) discontinued the trastuzumab-based chemotherapy due to disease progression, whereas three patients were still on treatment. These three patients belonged to the Homo-HER2-positive group. The median number of cycles of trastuzumab therapy was 23.7 (range 2–62) in the Homo-HER2-positive group and 9.3 (range 1–18) in the Hetero-HER2-positive group, respectively. Fifteen patients (53.6%) died at the data cutoff. Second-line chemotherapy after disease progression was given to all patients (11/11) in the Homo-HER2-positive group. Meanwhile, in the Hetero-HER2-positive group, 11 of 14 (78.6%) patients received second-line chemotherapy. Taxans as monotherapy or weekly paclitaxel with ramucirumab were mainly initiated as post-discontinuation therapy in the both groups.

**Fig. 2:**
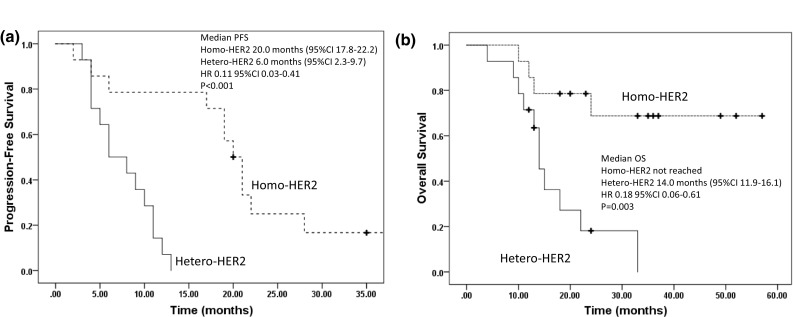
Progression-free survival and overall survival. Significant longer progression-free survival was seen in the Homo-HER2-positive group compared with the Hetero-HER2-positive group (**a**). Significant longer overall survival was seen in the Homo-HER2-positive group compared with the Hetero-HER2-positive group (**b**)

Survival is shown in Fig. 2. The median PFS in the Homo-HER2-positive group was significantly longer than that in the Hetero-HER2-positive group (20.0 months [95% CI 17.8–22.2] vs. 6.0 months [95% CI 2.3–9.7]; HR 0.11; 95% CI 0.03–0.41; *p* < 0.001; Fig. 2a). The median OS in the Homo-HER2-positive group was also significantly longer than that in the Hetero-HER2-positive group (not reached vs. 14.0 months [95% CI 11.9–16.1]; HR 0.18; 95% CI 0.06–0.61; *p *= 0.003; Fig. 2b). Platinum-based chemotherapy was also associated with both in PFS and OS in this cohort (Table 2). In the multivariate analysis stratified by HER2 heterogeneity and chemotherapy regimen, intratumoral HER2 heterogeneity remained significant both in PFS and OS (Table 3). In addition, because of small sample size, multivariate analyses were performed stratified by intratumoral HER2 heterogeneity and each single covariate chosen from all covariates. Intratumoral HER2 heterogeneity was the only independent predictor for PFS and OS (data not shown).

**Table 2:** Survival by univariate analysis

Characteristics (*n*)	mPFS (95% CI)	HR (95% CI)	*p* values	mOS (95% CI)	HR (95% CI)	*p* values
Median age (years)			0.373			0.124
≥ 68 (15)	9.0 (3.3–14.7)	1 (Reference)		18.0 (9.2–26.8)	1 (Reference)	
< 68 (13)	17.0 (9.2–24.8)	0.71 (0.32–1.56)		NR	0.44 (0.15–1.30)	
Gender			0.143			0.323
Male (21)	10.0 (5.5–14.5)	1 (Reference)		22.0 (7.5–36.5)	1 (Reference)	
Female (7)	19.0 (0.0–39.5)	0.49 (0.18–1.33)		NR	0.48 (0.11–2.14)	
ECOG PS			0.865			0.936
0 (16)	11.0 (5.1–16.9)	1 (Reference)		24.0 (4.7–43.3)	1 (Reference)	
1–2 (12)	10.0 (1.5–18.5)	1.07 (0.48–2.39)		22.0 (3.6–40.4)	1.04 (0.37–2.94)	
Tumor site			0.750			0.655
GEJ (8)	13.0 (6.1–19.9)	1 (Reference)		33.0 (4.1–61.9)	1 (Reference)	
Stomach (20)	9.0 (0.2–17.8)	1.15 (0.48–2.73)		22.0 (9.6–34.4)	1.25 (0.41–4.08)	
Differentiation			0.737			0.550
Differentiated type (23)	11.0 (7.9–14.1)	1 (Reference)		24.0 (4.3–43.7)	1 (Reference)	
Undifferentiated type (5)	9.0 (2.6–15.4)	1.20 (0.40–3.58)		15.0 (10.7–19.3)	1.48 (0.40–5.48)	
Visceral metastasis			0.116			0.271
Yes (15)	8.0 (3.3–12.7)	1 (Reference)		15.0 (11.5–18.5)	1 (Reference)	
No (13)	13.0 (3.6–22.4)	0.54 (0.24–1.21)		33.0 (NA)	0.56 (0.20–1.60)	
Adjuvant chemotherapy			0.856			0.702
Yes (17)	11.0 (5.6–16.4)	1 (Reference)		33.0 (0.6–65.4)	1 (Reference)	
No (11)	10.0 (0.0–24.0)	0.93 (0.42–2.08)		22.0 (11.2–32.8)	1.22 (0.44–3.37)	
Platinum-based						
Yes (25)	12.0 (8.3–15.7)	1 (Reference)	0.012	24.0 (5.1–42.9)	1 (Reference)	0.025
No (3)	4.0 (NA)	4.69 (1.18–18.65)		11.0 (7.8–14.2)	5.64 (1.02–31.38)	
HER2 status						
IHC 3+ (26)	11.0 (6.0–16.0)	1 (Reference)	0.283	22.0 (9.4–34.6)	1 (Reference)	0.273
IHC 2+/FISH positive (2)	5.0 (NA)	2.17 (0.49–9.63)		NR	0.04 (0.0–251.4)	
HER2 heterogeneity						
Yes (14)	6.0 (2.3–9.7)	1 (Reference)	<0.001	14.0 (11.9–16.1)	1 (Reference)	0.003
No (14)	20.0 (17.8–22.2)	0.11 (0.03–0.41)		NR	0.18 (0.06–0.61)	
CEA						
≥ 5.0 ng/ml (15)	6.0 (0.0–12.3)	1 (Reference)	0.659	22.0 (8.6–35.4)	1 (Reference)	0.804
< 5.0 ng/ml (13)	12.0 (5.0–19.1)	0.84 (0.38–1.85)		24.0 (1.2–46.9)	0.88 (0.32–2.43)	
CA 19-9						
≥ 37.0 U/ml (13)	8.0 (2.4––13.6)	1 (Reference)	0.627	18.0 (9.9–26.1)	1 (Reference)	0.641
< 37.0 U/ml (15)	11.0 (2.2–19.8)	0.82 (0.37–1.81)		24.0 (NA)	0.64 (0.23–1.77)	

*GEJ* gastroesophageal junction cancer, *ECOG PS* Eastern Cooperative Oncology Group Performance Status, *HR* hazard ratio, *95% CI* 95% confidential interval, *mPFS* median progression-free survival, *mOS* median overall survival, *NA* not applicable, *NR* not reached, *CEA* carcinoembryonic antigen, *CA 19-9* carbohydrate antigen 19-9

**Table 3:** Survival outcomes by multivariate analysis

Covariates	PFS		OS	
	HR 95% (CI)	*p* values	HR 95% (CI)	*p* values
Platinum-based				
Yes (25)	1 (Reference)	0.232	1 (Reference)	0.207
No (3)	2.34 (0.58–9.37)		3.06 (0.54–17.39)	
HER2 heterogeneity				
Yes (14)	1 (Reference)	0.002	1 (Reference)	0.013
No (14)	0.12 (0.03–0.46)		0.21 (0.06–0.72)	

*HR* hazard ratio, *95% CI* 95% confidential interval, *PFS* progression-free survival, *OS* overall survival

Because our data showed shorter survival of Hetero-HER2-positive group, it is unclear whether patients with Hetero-HER2-positive gastric cancer benefit from additional trastuzumab. Next, we compared survival of the Hetero-HER2-positive group and those of HER2-negative gastric cancer who received cisplatin and capecitabine. Eighteen patients with HER2-negative gastric cancer received cisplatin and capecitabine in the same period. The median PFS and OS were 5.7 months (95% CI 0.0–11.4) and 14.1 months (95% CI 6.6–21.6), respectively. No statistical difference was found between the Hetero-HER2-positive and HER2-negative group (HR 1.21; 95% CI 0.59–2.50; *p *= 0.598 for PFS and HR 0.97; 95% CI 0.44–2.13; *p *= 0.931 for OS), respectively, although the sample number is small (Fig S1).

**Fig. 3:**
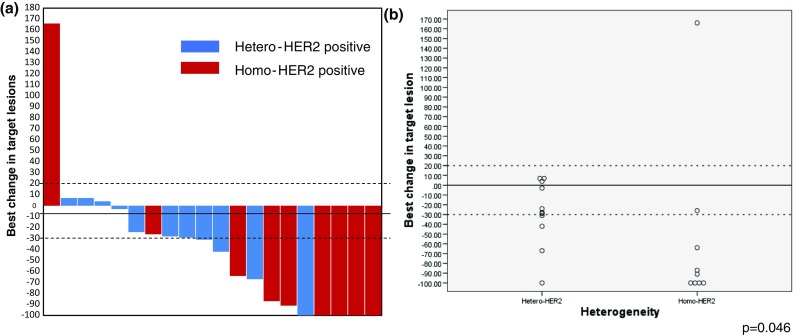
Best change from baseline in size of target lesion. Water-fall plot shows that patients in the Homo-HER2-positive group obtain deeper tumor shrinkage compared with the Hetero-HER2-positive group (**a**). Scatter plot shows statistically significant difference in tumor shrinkage between two groups (*p* = 0.046) (**b**)

Twenty patients had measurable lesions and best overall responses in these patients by RECIST were complete response in 5 patients (25%), partial response in 6 patients (30%), stable disease in 8 patients (40%), and progression disease in 1 patients (5%). ORR was 55.0% and disease control rate was 95.0%. A higher response rate was seen in the Homo-HER2-positive group than in the Hetero-HER2-positive group (77.8 vs. 36.4%; *p* = 0.092; Table 4). Moreover, deeper response was seen in the Homo-HER2-positive group compared with the Hetero-HER2-positive group (*p* = 0.046; Fig. 3).

**Table 4:** Best overall response

	Hetero-HER2 (%)	Homo-HER2 (%)	*p* values
	*n* = 12	*n* = 8	
CR	1 (8.3)	2 (25.0)	
PR	5 (41.7)	5 (62.5)	
SD	6 (50.0)	0 (0)	
PD	0 (0)	1 (12.5)	
ORR	6/12 (50.0)	7/8 (87.5)	0.282

*CR* complete response, *PR* partial response, *SD* stable disease, *PD* progression disease, *ORR* objective response rate, *Hetero-HER2* heterogeneously HER2 positive, *Homo-HER2* homogeneously HER2 positive

### Comparison of HER2 heterogeneity between surgical and biopsy specimens

Since the majority of patients who receive trastuzumab-based chemotherapy are unresectable, we examined whether biopsy specimens are useful to determine HER2 heterogeneity and to predict treatment efficacy. With respect to the definition of HER2 heterogeneity, the same as surgical specimen, if all tumor cells overexpressed HER2 protein with IHC 3+ or 2+ on all biopsy specimens, the tumor was defined as the Homo-HER2-positive group. The others were defined as the Hetero-HER2-positive group. Biopsy specimens were available for 25 patients (89%). Two of them were HER2 negative by examination of biopsy specimens, and these two patients belonged to the Hetero-HER2-positive group determined by surgical specimens. Comparison of HER2 heterogeneity between biopsy and surgical specimens is shown in Table S2. All Hetero-HER2-positive patients determined by surgical specimens were judged as Hetero-HER2 positive by biopsy specimens, whereas 2 of 12 patients determined as Homo-HER2 positive by surgical specimens were judged as Hetero-HER2 positive by biopsy specimens. Concordance rate of HER2 heterogeneity between surgical and biopsy specimens was 92%. Sensitivity, specificity, positive predict value, and negative predict value of evaluation biopsy specimens were 100, 83.3, 100, and 86.7%, respectively. With respect to survival, similar results to surgical specimens were shown: Homo-HER2-positive group determined by biopsy specimens showed longer PFS (20.0 months [95% CI 17.9–22.1] vs. 8.0 months [95% CI 4.2–11.8]; HR 0.18; 95% CI 0.06–0.54; *p* = 0.002) and OS (not reached vs. 14.0 months [95% CI 12.4–15.6]; HR 0.09; 95% CI 0.02–0.44; *p *= 0.003; Fig S2).

## Discussion

In this study, we carefully evaluated intratumoral HER2 heterogeneity using surgical specimens and examined predictive impact on trastuzumab efficacy. Our results showed, similar to breast cancer, patients with homogeneously HER2-positive gastric cancer had significantly longer survival than those with heterogeneously HER2-positive gastric cancer. In addition, higher response rate and deeper tumor shrinkage were found in the Homo-HER2-positive group. To our knowledge, this is the first report describing the clinical impact of intratumoral HER2 heterogeneity examined in surgical specimens on trastuzumab efficacy in patients with HER2-positive gastric cancer.

There have been several reports regarding intratumoral HER2 heterogeneity in gastric cancer [17–21]. According to these reports, HER2 heterogeneity was associated with IHC 2+ and diffuse histology. The incidences of intratumoral HER2 heterogeneity evaluated by IHC have been widely reported with a frequency from 39.0 to 75.4%. Kurokawa et al. reported the highest frequency of HER2 heterogeneity of 75.4% using whole-tissue sections when a cutoff value was set at 90% [18]. Meanwhile, screening data from the ToGA study, in which approximately 70% of the samples were biopsy specimens, showed that HER2 heterogeneity was found in 50.3% of the patients when the cutoff value was set at 30% [21]. Various cutoff values for HER2 heterogeneity assessment and different types of samples could affect the widely different frequencies of intratumoral HER2 heterogeneity in gastric cancer. In this study, we examined whole-layer surgical specimens and set the cutoff value as 100% in a representative section. In addition, our data demonstrated not only higher concordance rate of HER2 heterogeneity between surgical and biopsy specimens but similar predictive impact to surgical specimens, suggesting the utility of predictive impact of HER2 heterogeneity using biopsy specimen in unresectable HER2-positive gastric cancer.

In this study, patients in the Homo-HER2-positive group obtained greater benefit from trastuzumab-based chemotherapy compared with those in the Hetero-HER2 group. Previous data showed that homogeneous HER2 protein overexpression was associated with higher gene amplification by FISH [17]. In addition, recent reports demonstrated that higher *HER2* amplification level was associated with favorable trastuzumab efficacy [25, 26]. These reports are compatible with our survival results. Moreover, comprehensive genomic alternation and protein overexpression analysis revealed that cases with high-level receptor tyrosine kinase (RTK) amplification showed simple tumor biology such as corresponding protein overexpression and, rarely, other gene amplification [27]. Therefore, homogeneous HER2-positive gastric cancer may link to simple tumor biology, which means that almost all tumor cells depend on the HER2-driven pathway and these patients are probably the optimal population for anti-HER2 agent treatment. On the other hand, patients in the Hetero-HER2-positive group did not obtain enough survival benefit from trastuzumab. Previous data demonstrated that HER2 heterogeneity was involved in co-amplification or co-overexpression of other RTKs such as EGFR, cMET, and FGFR2 [27–30]. These molecular diversities may result in trastuzumab resistance, suggesting the requirement of new treatment strategy. Multikinase inhibitors or combination treatment corresponding to molecular alternations possibly overcome molecular diversity in gastric cancer [31, 32].

The mechanism of HER2 heterogeneity remains unclear. Lee et al. examined HER2 heterogeneity individually by IHC and FISH in the same area, and they revealed a strong correlation between HER2 protein over-expression and gene amplification. These data suggest that genetic heterogeneity is underling in HER2 heterogeneity [17]. In addition, several data showed that HER2 heterogeneity was associated with diffuse histology. Recent data from the cancer genome atlas proposed that gastric cancer was classified into four subtypes: Epstein–Barr virus positive, microsatellite unstable, chromosomal instability, and genomically stable tumor. Genomically stable tumors were enriched for diffuse histology and were associated with less frequency of RTKs amplification [30]. Because gastric cancer commonly showed histological heterogeneity, this histological heterogeneity may be related to genetic heterogeneity. Further molecular analysis in order to elucidate genetic heterogeneity in gastric cancer is warranted.

Unlike in breast cancer, the prognostic impact of HER2 is still controversial in gastric cancer [9, 18]. In addition, HER2 targeted agents, such as trastuzumab, lapatinib, T-DM1, and pertuzumab, have all been successfully developed in breast cancer, while all these anti-HER2 agents, except for trastuzumab, failed to show clinical benefit in gastric cancer [33–36]. Intratumoral HER2 heterogeneity may have influenced the inconsistencies in these results between breast and gastric cancers despite their having the same HER2 property: the median proportion with *HER2* amplification by FISH was 87.8% in breast cancer, whereas, in gastric cancer, almost half of the patients with HER2 protein overexpression exhibited it in less than 30% of the tumor cells [21, 23]. For future clinical trials for anti-HER2 agents in gastric cancer, consideration should be given not only to whether a tumor is HER2 positive but also to the degree in which the tumor depends on the HER2-driven pathway.

Limitations of this study include its retrospective nature from a single institution. In addition, the sample size is too small to conclude a clinical significance and the optimal cut-off value for trastuzumab. Nevertheless, our results would give credence and set a precedent for future research. In conclusion, intratumoral HER2 heterogeneity showed a clinical impact on trastuzumab efficacy in gastric cancer. These findings should be validated by larger independent cohorts and further molecular correlative analyses are warranted.

## Electronic supplementary material

Below is the link to the electronic supplementary material.

Supplementary material 1 (PPTX 61 kb)

Supplementary material 2 (PPTX 58 kb)

Supplementary material 3 (XLSX 12 kb)

Supplementary material 4 (XLSX 10 kb)

## Abbreviations

CEP17: Centromeric probe 17
FISH: Fluorescence in situ hybridization
HER2: Human epidermal growth factor receptor 2
Homo-HER2 positive: Homogeneously HER2 positive
Hetero-HER2 positive: Heterogeneously HER2 positive
IHC: Immunohistochemistry
PFS: Progression-free survival
ORR: Objective response rates
OS: Overall survival
RTK: Receptor tyrosine kinase

## References

[1] GLOBOCAN. 2012. http://globocan.iarc.fr/Default.aspx.

[2] Nonaka S, Oda I, Nakaya T (2011). Clinical impact of a strategy involving endoscopic submucosal dissection for early gastric cancer: determining the optimal pathway. Gastric Cancer.

[3] Sakuramoto S, Sasako M, Yamaguchi T (2007). Adjuvant chemotherapy for gastric cancer with S-1, an oral fluoropyrimidine. N Engl J Med.

[4] Cunningham D, Allum WH, Stenning SP (2006). Perioperative chemotherapy versus surgery alone for resectable gastroesophageal cancer. N Engl J Med.

[5] Macdonald JS, Smalley SR, Benedetti J (2001). Chemoradiotherapy after surgery compared with surgery alone for adenocarcinoma of the stomach or gastroesophageal junction. N Engl J Med.

[6] Cunningham D, Starling N, Rao S (2008). Capecitabine and oxaliplatin for advanced esophagogastric cancer. N Engl J Med.

[7] Van Cutsem E, Moiseyenko VM, Tjulandin S (2006). Phase III study of docetaxel and cisplatin plus fluorouracil compared with cisplatin and fluorouracil as first-line therapy for advanced gastric cancer: a report of the V325 Study Group. J Clin Oncol.

[8] Stern DF, Heffernan PA, Weinberg RA (1986). p185, a product of the neu proto-oncogene, is a receptor-like protein associated with tyrosine kinase activity. Mol Cell Biol.

[9] Terashima M, Kitada K, Ochiai A (2012). Impact of expression of human epidermal growth factor receptors EGFR and ERBB2 on survival in stage II/III gastric cancer. Clin Cancer Res.

[10] Okines AF, Thompson LC, Cunningham D (2013). Effect of HER2 on prognosis and benefit from peri-operative chemotherapy in early oesophago-gastric adenocarcinoma in the MAGIC trial. Ann Oncol.

[11] Gordon MA, Gundacker HM, Benedetti J (2013). Assessment of HER2 gene amplification in adenocarcinomas of the stomach or gastroesophageal junction in the INT-0116/SWOG9008 clinical trial. Ann Oncol.

[12] Slamon DJ, Leyland-Jones B, Shak S (2001). Use of chemotherapy plus a monoclonal antibody against HER2 for metastatic breast cancer that overexpresses HER2. N Engl J Med.

[13] Romond EH, Perez EA, Bryant J (2005). Trastuzumab plus adjuvant chemotherapy for operable HER2-positive breast cancer. N Engl J Med.

[14] Bang YJ, Van Cutsem E, Feyereislova A (2010). Trastuzumab in combination with chemotherapy versus chemotherapy alone for treatment of HER2-positive advanced gastric or gastro-oesophageal junction cancer (ToGA): a phase 3, open-label, randomised controlled trial. Lancet.

[15] Kurokawa Y, Sugimoto N, Miwa H (2014). Phase II study of trastuzumab in combination with S-1 plus cisplatin in HER2-positive gastric cancer (HERBIS-1). Br J Cancer.

[16] Miura Y, Takano T, Sukawa Y (2015). A phase II trial of 5-weekly S-1 plus cisplatin (CDDP) combination with trastuzumab (Tmab) for HER2-positive advanced gastric or esophagogastric junction (EGJ) cancer: WJOG 7212G (T-SPACE) study. J Clin Oncol.

[17] Lee HE, Park KU, Yoo SB (2013). Clinical significance of intratumoral HER2 heterogeneity in gastric cancer. Eur J Cancer.

[18] Kurokawa Y, Matsuura N, Kimura Y (2014). Multicenter large-scale study of prognostic impact of HER2 expression in patients with resectable gastric cancer. Gastric Cancer.

[19] Kim KC, Koh YW, Chang HM (2011). Evaluation of HER2 protein expression in gastric carcinomas: comparative analysis of 1,414 cases of whole-tissue sections and 595 cases of tissue microarrays. Ann Surg Oncol.

[20] Nishida Y, Kuwata T, Nitta H (2014). A novel gene-protein assay for evaluating HER2 status in gastric cancer: simultaneous analyses of HER2 protein overexpression and gene amplification reveal intratumoral heterogeneity. Gastric Cancer.

[21] Van Cutsem E, Bang YJ, Feng-Yi F (2014). HER2 screening data from ToGA: targeting HER2 in gastric and gastroesophageal junction cancer. Gastric Cancer.

[22] Hofmann M, Stoss O, Shi D (2008). Assessment of a HER2 scoring system for gastric cancer: results from a validation study. Histopathology.

[23] Lee HJ, Seo AN, Kim EJ (2014). HER2 heterogeneity affects trastuzumab responses and survival in patients with HER2-positive metastatic breast cancer. Am J Clin Pathol.

[24] Xu C, Liu Y, Jiang D (2017). Poor efficacy response to trastuzumab therapy in advanced gastric cancer with homogeneous HER2 positive and non-intestinal type. OncoTarget.

[25] Gomez-Martin C, Plaza JC, Pazo-Cid R (2013). Level of HER2 gene amplification predicts response and overall survival in HER2-positive advanced gastric cancer treated with trastuzumab. J Clin Oncol.

[26] Ock CY, Lee KW, Kim JW (2015). Optimal patient selection for trastuzumab treatment in HER2-positive advanced gastric cancer. Clin Cancer Res.

[27] Kuboki Y, Yamashita S, Niwa T (2016). Comprehensive analyses using next-generation sequencing and immunohistochemistry enable precise treatment in advanced gastric cancer. Ann Oncol.

[28] Nagatsuma AK, Aizawa M, Kuwata T (2014). Expression profiles of HER2, EGFR, MET and FGFR2 in a large cohort of patients with gastric adenocarcinoma. Gastric Cancer.

[29] Tajiri R, Ooi A, Fujimura T (2014). Intratumoral heterogeneous amplification of ERBB2 and subclonal genetic diversity in gastric cancers revealed by multiple ligation-dependent probe amplification and fluorescence in situ hybridization. Hum Pathol.

[30] Cancer Genome Atlas Research Network (2014). N. Comprehensive molecular characterization of gastric adenocarcinoma. Nature.

[31] Pavlakis N, Sjoquist KM, Martin AJ (2016). Regorafenib for the treatment of advanced gastric cancer (INTEGRATE): a multinational placebo-controlled Phase II trial. J Clin Oncol.

[32] Yaeger R, Cercek A, O’Reilly EM (2015). Pilot trial of combined BRAF and EGFR inhibition in BRAF-mutant metastatic colorectal cancer patients. Clin Cancer Res.

[33] Hecht JR, Bang YJ, Qin SK (2016). Lapatinib in combination with capecitabine plus oxaliplatin in human epidermal growth factor receptor 2-positive advanced or metastatic gastric, esophageal, or gastroesophageal adenocarcinoma: TRIO-013/LOGiC—a randomized Phase III trial. J Clin Oncol.

[34] Satoh T, Xu RH, Chung HC (2014). Lapatinib plus paclitaxel versus paclitaxel alone in the second-line treatment of HER2-amplified advanced gastric cancer in Asian populations: TyTAN—a randomized, Phase III study. J Clin Oncol.

[35] Kang Y, Shah MA, Ohtsu A (2016). A randomized, open-label, multicenter, adaptive phase 2/3 study of trastuzumab emtansine (T-DM1) versus a taxane (TAX) in patients (pts) with previously treated HER2-positive locally advanced or metastatic gastric/gastroesophageal junction adenocarcinoma (LA/MGC/GEJC). J Clin Oncol..

[36] Tabernero J, Hoff P, Shen L, Ohtsu A, Shah M, Cheng K, et al. Pertuzumab + trastuzumab + chemotherapy for HER2-positive metastatic gastric or gastro-oesophageal junction cancer: final analysis of a Phase III study (JACOB) ESMO in Madrid Spain 2017. 10.1016/S1470-2045(18)30481-9 30217672

