# Supplementary figures and images for: Clinical impact of intratumoral HER2 heterogeneity on trastuzumab efficacy in patients with HER2-positive gastric cancer
Source: J Gastroenterol. 2018 Apr 9;53(11):1186–95. doi: 10.1007/s00535-018-1464-0 (PMC6209002; doi:10.1007/s00535-018-1464-0)

## Slide 1
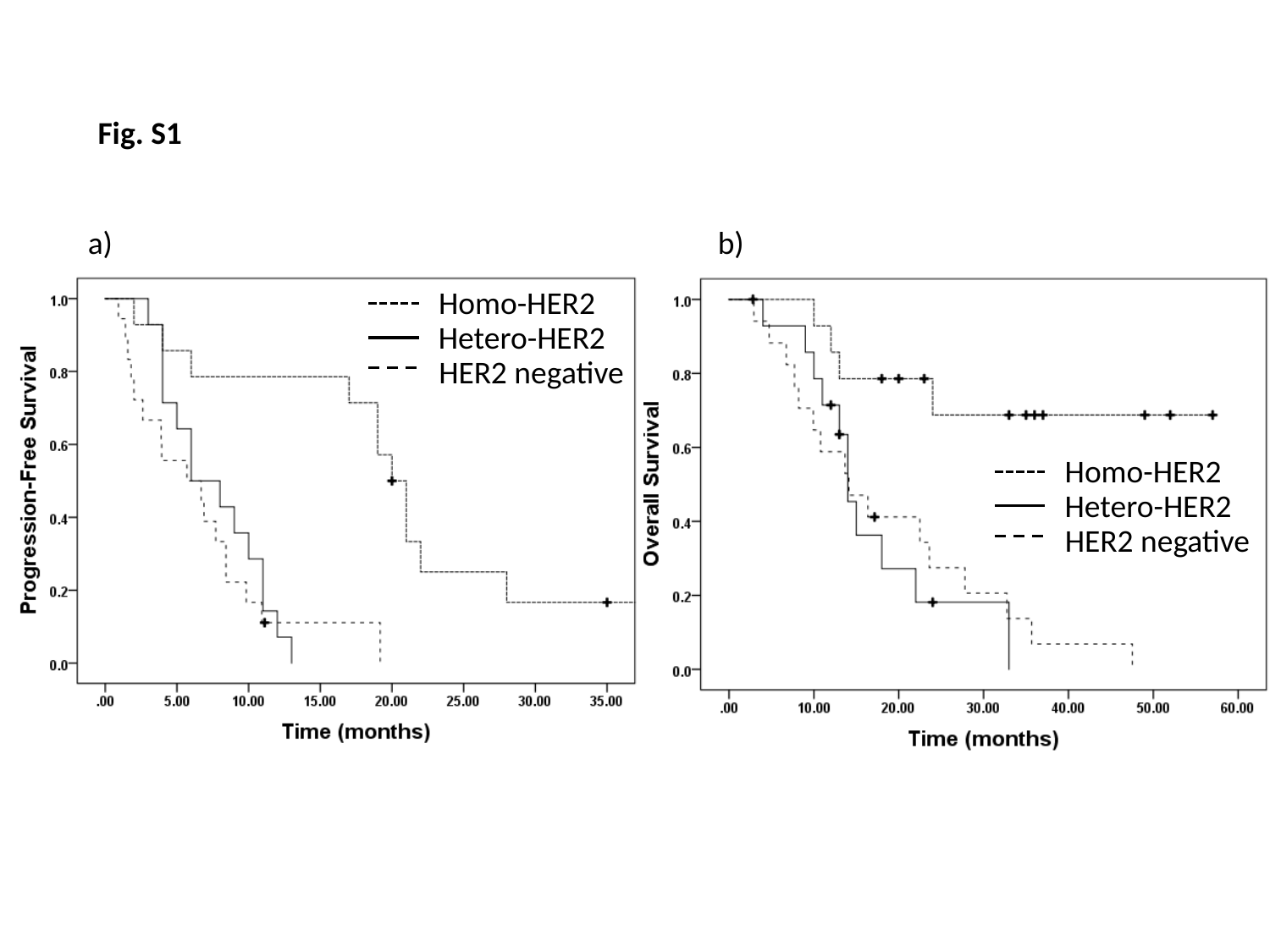

Fig. S1
a)
b)
Homo-HER2
Hetero-HER2
HER2 negative
Homo-HER2
Hetero-HER2
HER2 negative

Supplement: Supplementary file 1 — Supplementary material 1 (PPTX 61 kb) [file 535_2018_1464_MOESM1_ESM.pptx]
