# Supplementary material for: Clinical impact of intratumoral HER2 heterogeneity on trastuzumab efficacy in patients with HER2-positive gastric cancer
Source: J Gastroenterol. 2018 Apr 9;53(11):1186–95. doi: 10.1007/s00535-018-1464-0 (PMC6209002; doi:10.1007/s00535-018-1464-0)
Supplement: Supplementary file 2 — Supplementary material 2 (PPTX 58 kb) [file 535_2018_1464_MOESM2_ESM.pptx]

## Slide 1
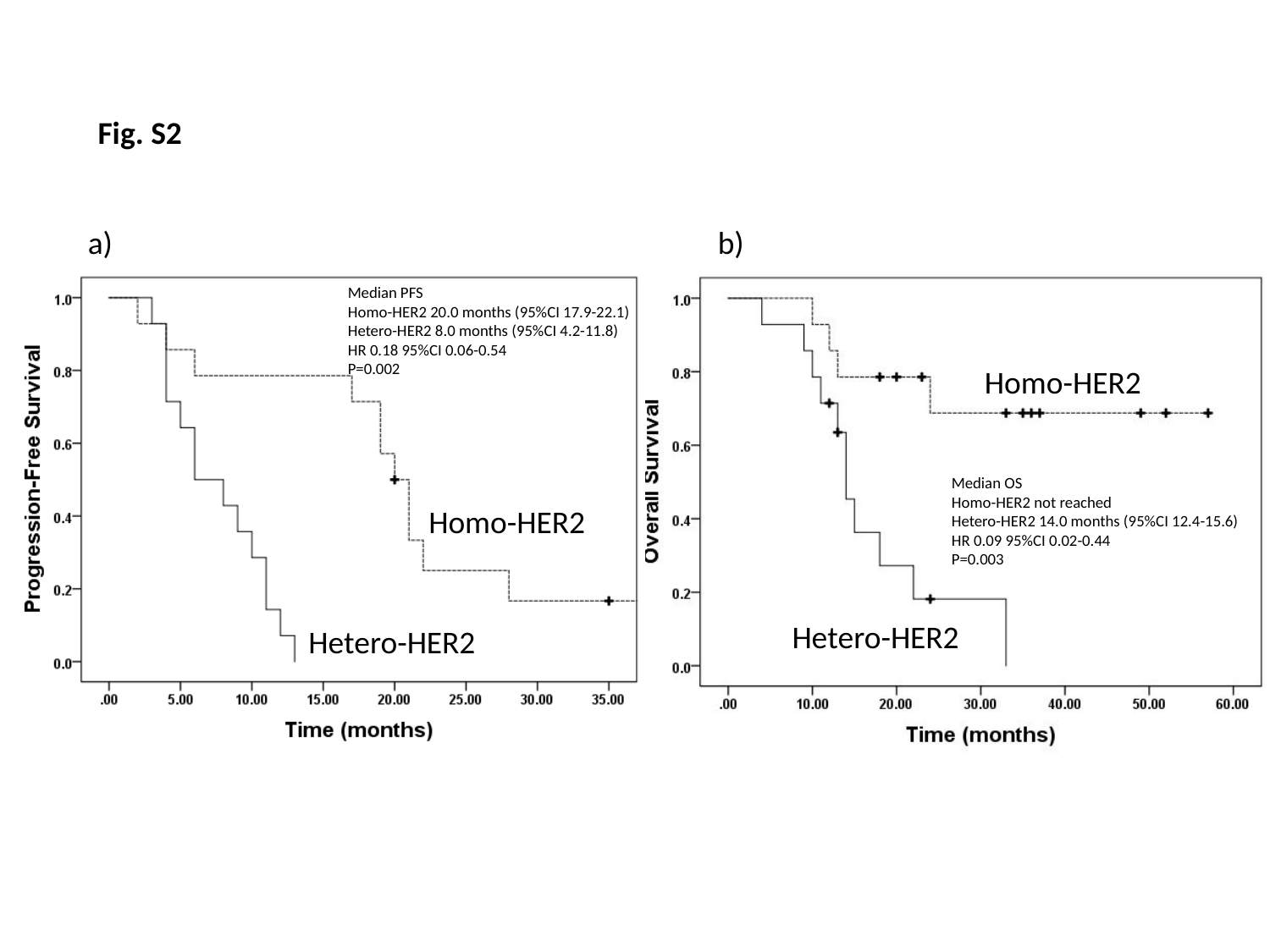

Fig. S2
a)
b)
Median PFS
Homo-HER2 20.0 months (95%CI 17.9-22.1)
Hetero-HER2 8.0 months (95%CI 4.2-11.8)
HR 0.18 95%CI 0.06-0.54
P=0.002
Homo-HER2
Median OS
Homo-HER2 not reached
Hetero-HER2 14.0 months (95%CI 12.4-15.6)
HR 0.09 95%CI 0.02-0.44
P=0.003
Homo-HER2
Hetero-HER2
Hetero-HER2
